# Propyl 2-(5-bromo-3-methyl­sulfinyl-1-benzofuran-2-yl)acetate

**DOI:** 10.1107/S160053680900453X

**Published:** 2009-02-13

**Authors:** Hong Dae Choi, Pil Ja Seo, Byeng Wha Son, Uk Lee

**Affiliations:** a Department of Chemistry, Dongeui University, San 24 Kaya-dong Busanjin-gu, Busan 614-714, Republic of Korea; b Department of Chemistry, Pukyong National University, 599-1 Daeyeon 3-dong Nam-gu, Busan 608-737, Republic of Korea

## Abstract

In the title compound, C_14_H_15_BrO_4_S, the S atom has a distorted trigonal–pyramidal coordination. The O atom and the methyl group of the methyl­sulfinyl substituent lie on opposite sides of the plane of the benzofuran fragment. The mol­ecules form slightly slipped π-stacked inversion-symmetric dimers by inter­molecular aromatic π–π inter­actions, with a centroid-to-centroid distance of 3.695 (4) Å between the benzene rings of neighbouring mol­ecules. The crystal packing is further stabilized by inter­molecular C—H⋯π inter­actions between the methyl­ene H atoms of the propyl group towards the benzene and furan rings of neighbouring mol­ecules, respectively. Additionally, the crystal structure exhibits weak inter­molecular C—H⋯O hydrogen bonds.

## Related literature

For the crystal structures of similar alkyl 2-(5-bromo-3-methyl­sulfinyl-1-benzofuran-2-yl)acetate derivatives, see: Choi *et al.* (2008*a* ▶,*b* ▶).
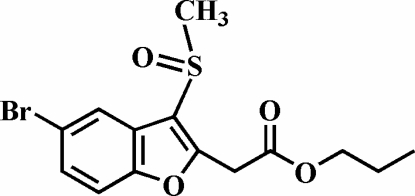


## Experimental

### 

#### Crystal data

C_14_H_15_BrO_4_S
*M* *_r_* = 359.23
Triclinic, 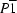

*a* = 8.4538 (6) Å
*b* = 9.8823 (7) Å
*c* = 10.3231 (7) Å
α = 72.358 (1)°
β = 81.200 (1)°
γ = 65.443 (1)°
*V* = 747.16 (9) Å^3^
*Z* = 2
Mo *K*α radiation
μ = 2.90 mm^−1^
*T* = 298 K
0.60 × 0.50 × 0.20 mm

#### Data collection

Bruker SMART CCD diffractometer
Absorption correction: multi-scan (*SADABS*; Sheldrick, 1999 ▶) *T*_min_ = 0.187, *T*_max_ = 0.556
3932 measured reflections
2593 independent reflections
2359 reflections with *I* > 2σ(*I*)
*R*_int_ = 0.014

#### Refinement

*R*[*F* ^2^ > 2σ(*F* ^2^)] = 0.034
*wR*(*F* ^2^) = 0.097
*S* = 1.07
2593 reflections
182 parameters
H-atom parameters constrained
Δρ_max_ = 0.62 e Å^−3^
Δρ_min_ = −0.38 e Å^−3^

### 

Data collection: *SMART* (Bruker, 2001 ▶); cell refinement: *SAINT* (Bruker, 2001 ▶); data reduction: *SAINT*; program(s) used to solve structure: *SHELXS97* (Sheldrick, 2008 ▶); program(s) used to refine structure: *SHELXL97* (Sheldrick, 2008 ▶); molecular graphics: *ORTEP-3* (Farrugia, 1997 ▶) and *DIAMOND* (Brandenburg, 1998 ▶); software used to prepare material for publication: *SHELXL97*.

## Supplementary Material

Crystal structure: contains datablocks global, I. DOI: 10.1107/S160053680900453X/zl2167sup1.cif

Structure factors: contains datablocks I. DOI: 10.1107/S160053680900453X/zl2167Isup2.hkl

Additional supplementary materials:  crystallographic information; 3D view; checkCIF report

## Figures and Tables

**Table 1:** Hydrogen-bond geometry (Å, °)

*D*—H⋯*A*	*D*—H	H⋯*A*	*D*⋯*A*	*D*—H⋯*A*
C11—H11*B*⋯*Cg*1^i^	0.97	3.02	3.720 (3)	130
C12—H12*B*⋯*Cg*2^i^	0.97	2.90	3.826 (3)	161
C3—H3⋯O4^ii^	0.93	2.54	3.424 (3)	159
C5—H5⋯O3^iii^	0.93	2.58	3.430 (4)	152
C9—H9*B*⋯O4^iv^	0.97	2.37	3.321 (3)	167

Symmetry codes: (i) 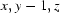; (ii) 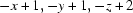; (iii) 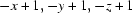; (iv) 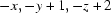. *Cg*1 and *Cg*2 are the centroids of the C2–C7 benzene ring and the C1/C2/C7/O1/C8 furan ring, respectively.

**Fig. 1.**
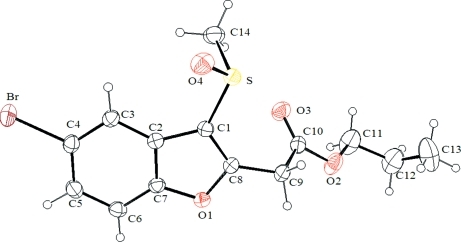
The molecular structure of the title compound, showing displacement ellipsoids drawn at the 30% probability level.

**Fig. 2.**
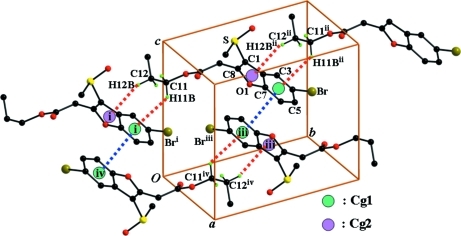
π—π and C—H···π interactions (dotted lines) in the title compound. Cg denotes the ring centroids. [Symmetry codes: (i) x, y-1, z; (ii) x, y+1, z; (iii) -x+1, -y+1, -z+1; (iv) -x+1, -y, -z+1.]

**Fig. 3.**
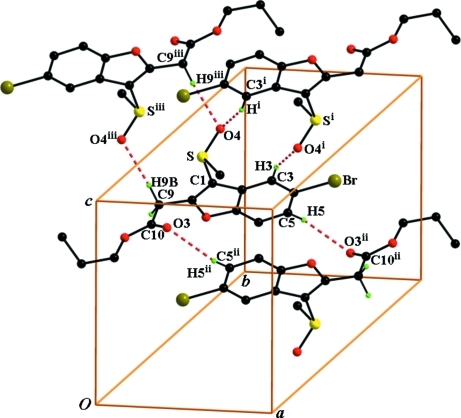
Intermolecular C—H···O hydrogen bonds (dotted lines) in the title compound. [Symmetry codes: (i) -x+1, -y+1, -z+2; (ii) -x+1, -y+1, -z+1; (iii) -x, -y+1, -z+2.]

## supplementary crystallographic information

### Comment

As a part of our ongoing research on the synthesis and structure of alkyl 2-(5-bromo-3-methylsulfinyl-1-benzofuran-2-yl)acetate analogues, we have recently described the crystal structures of isopropyl 2-(5-bromo-3-methylsulfinyl-1-benzofuran-2-yl)acetate (Choi *et al.*, 2008*a*) and methyl 2-(5-bromo-3-methylsulfinyl-1-benzofuran-2-yl)acetate (Choi *et al.*, 2008*b*). Here we report the crystal structure of the title compound, propyl 2-(5-bromo-3-methylsulfinyl-1-benzofuran-2-yl)acetate (Fig. 1).

The benzofuran unit is essentially planar, with a mean deviation of 0.013 (2) Å from the least-squares plane defined by the nine constituent atoms. The molecular packing (Fig. 2) is stabilized by intermolecular π–π stacking interactions between the benzene rings of neighbouring molecules. Via this interaction the molecules form slightly slipped π-stacked inversion symmetric dimers, with a centroid–centroid distance *Cg*1···*Cg*1^iii^ of 3.695 (4) Å between the benzene rings of neighbouring molecules. (*Cg* is the centroid of the C2–C7 benzene ring, symmetry code as in Fig. 2). The molecular packing is further stabilized by C—H···π interactions; one between the hydrogen of the C11-methylene group and the benzene ring of the benzofuran unit, with a C11—H11B···*Cg*1^i^ separation of 3.02 Å, and a second between the hydrogen of the C12-methylene group and the furan ring of the benzofuran unit, with a C12—H12B···*Cg*2^i^ separation of 2.90 Å (Table 1 and Fig. 2; *Cg*1 and *Cg*2 are the centroids of the C2–C7 benzene ring and the C1/C2/C7/O1/C8 furan ring, respectively, symmetry code as in Fig. 2). In addition, three weak intermolecular C—H···O hydrogen bonds in the structure were observed (Table 1 and Fig. 3); one between the hydrogen on benzene ring and the oxygen of the S═O unit (C3—H3···O4^ii^), a second between the hydrogen on benzene ring and the oxygen of the C═O unit (C5—H5···O3^iii^), and a third between the hydrogen of the C9–methylene group and the S═O unit (C9—H9B···O4^iv^), respectively.

### Experimental

77% 3-chloroperoxybenzoic acid (179 mg, 0.8 mmol) was added in small portions to a stirred solution of propyl 2-(5-bromo-3-methylsulfanyl-1-benzofuran-2-yl)acetate (629 mg, 0.75 mmol) in dichloromethane (30 ml) at 273 K. After being stirred for 3 h at room temperature, the mixture was washed with saturated sodium bicarbonate solution and the organic layer was separated, dried over magnesium sulfate, filtered and concentrated in vacuum. The residue was purified by column chromatography (hexane–ethyl acetate, 1:2 v/v) to afford the title compound as a colorless solid [yield 81%, m.p. 413–413.5 K; *R*_f_ = 0.55 (hexane–ethyl acetate, 1:2 v/v)]. Single crystals suitable for X-ray diffraction were prepared by evaporation of a solution of the title compound in benzene at room temperature. Spectroscopic analysis: ^1^H NMR (CDCl_3_, 400 MHz) δ 0.94 (t, J = 7.32 Hz, 3H), 1.63–1.72 (m, 2H), 3.07 (s, 3H), 4.05 (s, 2H), 4.11 (t, J = 6.96 Hz, 2H), 7.40 (d, J = 8.76 Hz, 1H), 7.48 (dd, J = 8.76 Hz and J = 1.84 Hz, 1H), 8.09 (d, J = 1.84 Hz, 1H); EI-MS 360 [M+2], 358 [M^+^].

### Refinement

All H atoms were geometrically positioned and refined using a riding model, with C—H = 0.93 Å for the aryl, 0.97 Å for the methylene, and 0.96 Å for the methyl H atoms. *U*iso(H) = 1.2*U*eq(C) for the ary and methylene H atoms, and 1.5*U*eq(C) for methyl H atoms.

### Crystal data

**Table:**

C_14_H_15_BrO_4_S	*Z* = 2
*M**_r_* = 359.23	*F*(000) = 364
Triclinic, *P*1	*D*_x_ = 1.597 Mg m^−^^3^
Hall symbol: -p_1	Mo *K*α radiation, λ = 0.71073 Å
*a* = 8.4538 (6) Å	Cell parameters from 2872 reflections
*b* = 9.8823 (7) Å	θ = 2.7–28.0°
*c* = 10.3231 (7) Å	µ = 2.90 mm^−^^1^
α = 72.358 (1)°	*T* = 298 K
β = 81.200 (1)°	Block, colourless
γ = 65.443 (1)°	0.60 × 0.50 × 0.20 mm
*V* = 747.16 (9) Å^3^	

### Data collection

**Table:**

Bruker SMART CCD diffractometer	2593 independent reflections
Radiation source: fine-focus sealed tube	2359 reflections with *I* > 2σ(*I*)
graphite	*R*_int_ = 0.014
Detector resolution: 10.0 pixels mm^-1^	θ_max_ = 25.0°, θ_min_ = 2.4°
φ and ω scans	*h* = −9→10
Absorption correction: multi-scan (*SADABS*; Sheldrick, 1999)	*k* = −10→11
*T*_min_ = 0.187, *T*_max_ = 0.556	*l* = −12→10
3932 measured reflections	

### Refinement

**Table:**

Refinement on *F*^2^	Primary atom site location: structure-invariant direct methods
Least-squares matrix: full	Secondary atom site location: difference Fourier map
*R*[*F*^2^ > 2σ(*F*^2^)] = 0.034	Hydrogen site location: difference Fourier map
*wR*(*F*^2^) = 0.097	H-atom parameters constrained
*S* = 1.07	*w* = 1/[σ^2^(*F*_o_^2^) + (0.0598*P*)^2^ + 0.3564*P*] where *P* = (*F*_o_^2^ + 2*F*_c_^2^)/3
2593 reflections	(Δ/σ)_max_ < 0.001
182 parameters	Δρ_max_ = 0.62 e Å^−^^3^
0 restraints	Δρ_min_ = −0.38 e Å^−^^3^

### Special details

**Table:**

Geometry. All esds (except the esd in the dihedral angle between two l.s. planes) are estimated using the full covariance matrix. The cell esds are taken into account individually in the estimation of esds in distances, angles and torsion angles; correlations between esds in cell parameters are only used when they are defined by crystal symmetry. An approximate (isotropic) treatment of cell esds is used for estimating esds involving l.s. planes.
Refinement. Refinement of F^2^ against ALL reflections. The weighted R-factor wR and goodness of fit S are based on F^2^, conventional R-factors R are based on F, with F set to zero for negative F^2^. The threshold expression of F^2^ > 2sigma(F^2^) is used only for calculating R-factors(gt) etc. and is not relevant to the choice of reflections for refinement. R-factors based on F^2^ are statistically about twice as large as those based on F, and R- factors based on ALL data will be even larger.

### Fractional atomic coordinates and isotropic or equivalent isotropic displacement parameters (Å^2^)

**Table:**

	*x*	*y*	*z*	*U*_iso_*/*U*_eq_	
Br	0.68856 (4)	0.78591 (4)	0.61232 (4)	0.06138 (16)	
S	0.26099 (9)	0.40306 (8)	0.96326 (6)	0.04277 (19)	
O1	0.1656 (2)	0.5416 (2)	0.57292 (17)	0.0389 (4)	
O2	0.0410 (3)	0.1435 (2)	0.7276 (3)	0.0651 (6)	
O3	0.2856 (3)	0.1366 (3)	0.7909 (3)	0.0628 (6)	
O4	0.2583 (3)	0.5257 (3)	1.0205 (2)	0.0565 (5)	
C1	0.2557 (3)	0.4773 (3)	0.7850 (2)	0.0355 (5)	
C2	0.3457 (3)	0.5694 (3)	0.6968 (2)	0.0345 (5)	
C3	0.4701 (3)	0.6215 (3)	0.7124 (3)	0.0390 (6)	
H3	0.5166	0.5970	0.7965	0.047*	
C4	0.5206 (3)	0.7106 (3)	0.5974 (3)	0.0411 (6)	
C5	0.4525 (4)	0.7510 (3)	0.4691 (3)	0.0448 (6)	
H5	0.4882	0.8144	0.3949	0.054*	
C6	0.3327 (3)	0.6968 (3)	0.4533 (3)	0.0421 (6)	
H6	0.2877	0.7199	0.3689	0.051*	
C7	0.2826 (3)	0.6069 (3)	0.5677 (3)	0.0367 (5)	
C8	0.1513 (3)	0.4645 (3)	0.7068 (3)	0.0368 (5)	
C9	0.0363 (3)	0.3784 (3)	0.7351 (3)	0.0408 (6)	
H9A	−0.0412	0.4178	0.6600	0.049*	
H9B	−0.0347	0.3956	0.8164	0.049*	
C10	0.1380 (4)	0.2072 (3)	0.7546 (3)	0.0448 (6)	
C11	0.1221 (5)	−0.0227 (4)	0.7402 (6)	0.0860 (14)	
H11A	0.1843	−0.0769	0.8242	0.103*	
H11B	0.2040	−0.0434	0.6648	0.103*	
C12	−0.0195 (6)	−0.0757 (5)	0.7394 (6)	0.0900 (14)	
H12A	−0.0800	−0.0196	0.6548	0.108*	
H12B	0.0329	−0.1842	0.7418	0.108*	
C13	−0.1468 (9)	−0.0548 (7)	0.8527 (6)	0.122 (2)	
H13A	−0.2019	0.0527	0.8501	0.147*	
H13B	−0.0889	−0.1122	0.9372	0.147*	
H13C	−0.2332	−0.0913	0.8452	0.147*	
C14	0.4798 (4)	0.2618 (4)	0.9755 (3)	0.0575 (8)	
H14A	0.5592	0.3128	0.9465	0.086*	
H14B	0.4965	0.1959	0.9185	0.086*	
H14C	0.5011	0.2010	1.0681	0.086*	

### Atomic displacement parameters (Å^2^)

**Table:**

	*U*^11^	*U*^22^	*U*^33^	*U*^12^	*U*^13^	*U*^23^
Br	0.0581 (2)	0.0617 (2)	0.0766 (3)	−0.03898 (17)	−0.00471 (16)	−0.01173 (17)
S	0.0443 (4)	0.0525 (4)	0.0332 (3)	−0.0238 (3)	−0.0022 (3)	−0.0064 (3)
O1	0.0385 (9)	0.0443 (10)	0.0356 (9)	−0.0182 (8)	−0.0059 (7)	−0.0078 (8)
O2	0.0436 (11)	0.0429 (11)	0.114 (2)	−0.0170 (9)	−0.0113 (12)	−0.0243 (12)
O3	0.0443 (12)	0.0509 (12)	0.0865 (16)	−0.0157 (10)	−0.0153 (11)	−0.0066 (11)
O4	0.0607 (13)	0.0705 (14)	0.0457 (11)	−0.0259 (11)	0.0001 (10)	−0.0262 (10)
C1	0.0360 (12)	0.0366 (12)	0.0339 (12)	−0.0145 (10)	−0.0016 (10)	−0.0087 (10)
C2	0.0341 (12)	0.0333 (12)	0.0356 (12)	−0.0117 (10)	−0.0021 (10)	−0.0102 (10)
C3	0.0394 (13)	0.0395 (13)	0.0412 (14)	−0.0160 (11)	−0.0047 (10)	−0.0123 (11)
C4	0.0376 (13)	0.0365 (13)	0.0513 (16)	−0.0157 (11)	−0.0002 (11)	−0.0137 (11)
C5	0.0430 (14)	0.0394 (14)	0.0465 (15)	−0.0165 (12)	0.0019 (12)	−0.0049 (12)
C6	0.0429 (14)	0.0438 (14)	0.0348 (13)	−0.0143 (12)	−0.0048 (11)	−0.0060 (11)
C7	0.0335 (12)	0.0342 (12)	0.0409 (14)	−0.0108 (10)	−0.0047 (10)	−0.0097 (10)
C8	0.0340 (12)	0.0382 (13)	0.0373 (13)	−0.0142 (10)	−0.0016 (10)	−0.0084 (10)
C9	0.0366 (13)	0.0446 (14)	0.0449 (14)	−0.0186 (11)	−0.0029 (11)	−0.0123 (11)
C10	0.0401 (14)	0.0463 (15)	0.0491 (15)	−0.0210 (12)	−0.0001 (12)	−0.0089 (12)
C11	0.055 (2)	0.0450 (19)	0.158 (4)	−0.0120 (16)	−0.004 (2)	−0.039 (2)
C12	0.075 (3)	0.047 (2)	0.151 (4)	−0.0233 (18)	−0.005 (3)	−0.032 (2)
C13	0.138 (5)	0.095 (4)	0.140 (5)	−0.073 (4)	0.050 (4)	−0.026 (3)
C14	0.0550 (18)	0.0543 (18)	0.0555 (18)	−0.0142 (15)	−0.0160 (14)	−0.0075 (14)

### Geometric parameters (Å, °)

**Table:**

Br—C4	1.899 (3)	C6—C7	1.374 (4)
S—O4	1.492 (2)	C6—H6	0.9300
S—C1	1.763 (3)	C8—C9	1.486 (4)
S—C14	1.790 (3)	C9—C10	1.509 (4)
O1—C8	1.375 (3)	C9—H9A	0.9700
O1—C7	1.375 (3)	C9—H9B	0.9700
O2—C10	1.323 (4)	C11—C12	1.494 (6)
O2—C11	1.465 (4)	C11—H11A	0.9700
O3—C10	1.202 (4)	C11—H11B	0.9700
C1—C8	1.349 (4)	C12—C13	1.465 (7)
C1—C2	1.448 (4)	C12—H12A	0.9700
C2—C3	1.396 (4)	C12—H12B	0.9700
C2—C7	1.397 (3)	C13—H13A	0.9600
C3—C4	1.376 (4)	C13—H13B	0.9600
C3—H3	0.9300	C13—H13C	0.9600
C4—C5	1.402 (4)	C14—H14A	0.9600
C5—C6	1.377 (4)	C14—H14B	0.9600
C5—H5	0.9300	C14—H14C	0.9600
			
O4—S—C1	107.01 (13)	C10—C9—H9A	109.2
O4—S—C14	106.32 (14)	C8—C9—H9B	109.2
C1—S—C14	98.46 (14)	C10—C9—H9B	109.2
C8—O1—C7	106.53 (19)	H9A—C9—H9B	107.9
C10—O2—C11	117.2 (2)	O3—C10—O2	124.0 (3)
C8—C1—C2	107.4 (2)	O3—C10—C9	125.6 (3)
C8—C1—S	123.4 (2)	O2—C10—C9	110.4 (2)
C2—C1—S	129.00 (19)	O2—C11—C12	107.7 (3)
C3—C2—C7	119.3 (2)	O2—C11—H11A	110.2
C3—C2—C1	136.1 (2)	C12—C11—H11A	110.2
C7—C2—C1	104.6 (2)	O2—C11—H11B	110.2
C4—C3—C2	116.8 (2)	C12—C11—H11B	110.2
C4—C3—H3	121.6	H11A—C11—H11B	108.5
C2—C3—H3	121.6	C13—C12—C11	114.4 (5)
C3—C4—C5	123.3 (3)	C13—C12—H12A	108.7
C3—C4—Br	118.7 (2)	C11—C12—H12A	108.7
C5—C4—Br	117.9 (2)	C13—C12—H12B	108.7
C6—C5—C4	119.8 (3)	C11—C12—H12B	108.7
C6—C5—H5	120.1	H12A—C12—H12B	107.6
C4—C5—H5	120.1	C12—C13—H13A	109.5
C7—C6—C5	117.1 (2)	C12—C13—H13B	109.5
C7—C6—H6	121.5	H13A—C13—H13B	109.5
C5—C6—H6	121.5	C12—C13—H13C	109.5
O1—C7—C6	125.9 (2)	H13A—C13—H13C	109.5
O1—C7—C2	110.5 (2)	H13B—C13—H13C	109.5
C6—C7—C2	123.6 (2)	S—C14—H14A	109.5
C1—C8—O1	111.0 (2)	S—C14—H14B	109.5
C1—C8—C9	133.2 (2)	H14A—C14—H14B	109.5
O1—C8—C9	115.7 (2)	S—C14—H14C	109.5
C8—C9—C10	112.2 (2)	H14A—C14—H14C	109.5
C8—C9—H9A	109.2	H14B—C14—H14C	109.5
			
O4—S—C1—C8	−134.5 (2)	C3—C2—C7—O1	178.3 (2)
C14—S—C1—C8	115.5 (2)	C1—C2—C7—O1	−1.2 (3)
O4—S—C1—C2	40.5 (3)	C3—C2—C7—C6	−1.8 (4)
C14—S—C1—C2	−69.5 (3)	C1—C2—C7—C6	178.8 (2)
C8—C1—C2—C3	−178.6 (3)	C2—C1—C8—O1	0.0 (3)
S—C1—C2—C3	5.7 (4)	S—C1—C8—O1	175.96 (17)
C8—C1—C2—C7	0.7 (3)	C2—C1—C8—C9	175.7 (3)
S—C1—C2—C7	−174.95 (19)	S—C1—C8—C9	−8.3 (4)
C7—C2—C3—C4	1.3 (4)	C7—O1—C8—C1	−0.7 (3)
C1—C2—C3—C4	−179.5 (3)	C7—O1—C8—C9	−177.3 (2)
C2—C3—C4—C5	0.6 (4)	C1—C8—C9—C10	−73.6 (4)
C2—C3—C4—Br	179.65 (18)	O1—C8—C9—C10	102.0 (3)
C3—C4—C5—C6	−2.0 (4)	C11—O2—C10—O3	−1.5 (5)
Br—C4—C5—C6	178.9 (2)	C11—O2—C10—C9	179.2 (3)
C4—C5—C6—C7	1.5 (4)	C8—C9—C10—O3	24.7 (4)
C8—O1—C7—C6	−178.8 (2)	C8—C9—C10—O2	−156.0 (2)
C8—O1—C7—C2	1.2 (3)	C10—O2—C11—C12	166.2 (4)
C5—C6—C7—O1	−179.7 (2)	O2—C11—C12—C13	−62.0 (6)
C5—C6—C7—C2	0.3 (4)		

### Hydrogen-bond geometry (Å, °)

**Table:**

*D*—H···*A*	*D*—H	H···*A*	*D*···*A*	*D*—H···*A*
C11—H11B···Cg1^i^	0.97	3.02	3.720 (3)	130
C12—H12B···Cg2^i^	0.97	2.90	3.826 (3)	161
C3—H3···O4^ii^	0.93	2.54	3.424 (3)	159
C5—H5···O3^iii^	0.93	2.58	3.430 (4)	152
C9—H9B···O4^iv^	0.97	2.37	3.321 (3)	167

Symmetry codes: (i) *x*, *y*−1, *z*; (ii) −*x*+1, −*y*+1, −*z*+2; (iii) −*x*+1, −*y*+1, −*z*+1; (iv) −*x*, −*y*+1, −*z*+2.
